# Adjusting the Equity Lens: Gaps in Addressing Health Equity in State Chronic Disease Prevention

**DOI:** 10.1089/heq.2018.0075

**Published:** 2019-03-29

**Authors:** Amy A. Eyler, Cheryl A. Valko, Marti Macchi, Zarina Fershteyn, Stephanie L. Mazzucca, Carol A. Brownson, Andrew Lau, Ross C. Brownson

**Affiliations:** ^1^ Prevention Research Center, Brown School, Washington University in St. Louis, St. Louis, Missouri.; ^2^ National Association of Chronic Disease Directors, Washington, District of Columbia.; ^3^ Arcora Foundation, Seattle, Washington.; ^4^ Division of Public Health Sciences, Alvin J. Siteman Cancer Center, Washington University School of Medicine, Washington University in St. Louis, St. Louis, Missouri.

**Keywords:** chronic disease prevention, equity, public health

## Abstract

**Purpose:** Chronic diseases cause a significant proportion of mortality and morbidity in the United States, although risk factors and prevalence rates vary by population subgroups. State chronic disease prevention practitioners are positioned to address these issues, yet little is known about how health equity is being incorporated into their work. The purpose of this study was to explore perceptions of health equity in a sample of state chronic disease practitioners.

**Methods:** Participants were selected in conjunction with a related evaluation of the National Association of Chronic Disease Directors (NACDD) capacity-building and evidence-based efforts. Four states were chosen for study based on variance in capacity. Directors in each of the states were interviewed and using snowball sampling, 8–12 practitioner interviews were conducted in each state, digitally audio recorded and transcribed. Using a comparative coding technique, themes and analyses were developed.

**Results:** Comments from the practitioners fell into three main and inter-related categories. First, they discussed the varying degrees of integration of health equity in their work. The second theme was collaboration and the importance of working within and outside of departments, as well as with the community. The third theme related to measurement and the need for better data that can be used to garner support and measure impact.

**Conclusion:** Chronic disease practitioners can play an important role in achieving health equity. Integrating this work more fully into chronic disease prevention and health promotion, developing strategic partnerships, tracking efforts, and measuring impact will improve practice and ultimately population health.

## Introduction

Chronic diseases account for the majority of deaths in the United States and prevention efforts remain a vital component of public health.^1^ However, surveillance data show that mortality and prevalence rates for chronic diseases vary greatly among population subgroups. For example, in the United States, the 2015 rate of cerebrovascular mortality was significantly higher for non-Hispanic blacks (61.3%) compared with non-Hispanic whites (47.7%).^2^ Disparities also exist by income level. The 2012 national age-standardized adult diabetes prevalence in the lowest income group was over double that of the highest income group, 17.8% and 8%, respectively.^3^ Because of the increasing awareness of the disparate burden of these diseases based on social and structural factors related to race, income, gender, sexual orientation, and living conditions, there is an emerging interest in addressing these health disparities through state-level prevention strategies.^4–6^ Reducing health disparities and determinants of these disparities is the underlying principle of health equity,^7,8^ which has become a significant focus within federal, state, and local public health agencies.^9–11^ State chronic disease practitioners serve as a bridge between federal and local programs and are uniquely positioned to implement and evaluate the most effective strategies to address disease disparities of their state population.

Since 2001, health equity has been integrated into the public health ethics framework (American Public Health Association).^12^ To ensure distributive justice (a core component in this framework), there is a need to ask, “Are the benefits and burdens distributed fairly?” A term often used to describe this concept is *equity lens*. Applying an *equity lens* refers to the examination of who experiences the benefits and burdens of policies and programs as well as the basis for differential experiences.^6^

This lens has a wide focus. Over the past decade, achieving health equity has become the aim of many national^13,14^ and international^15^ public health programs. Health equity plays prominently in Healthy People 2020, which has as one of its four overarching goals to achieve health equity, eliminate disparities, and improve the health of all groups.^13^ Additionally, health equity indicators are included throughout the standards and measures of the Public Health Accreditation Board, and accreditation criteria explicitly require that the health department must document efforts to address health equity among populations in the health department's jurisdiction.^16^ Data from the National Association of Chronic Disease Directors (NACDD^5,11^) and the Association of State and Territorial Health Officials^9^ indicate the increasing integration of health equity into infrastructure, strategic planning, and major activities within state health departments.

Although state public health agencies are integrating health equity into their work at the organizational level, the extent to which these efforts are integrated varies greatly.^8^ In 2014, 81% of health departments reported having a full-time staff-equivalent resource working on health equity or minority health, but integration of health equity within state chronic disease prevention needs improvement.^9^ A 2018 survey found that only 11% of state-level chronic disease practitioners agreed that health equity fell within their purview.^5^ Results from another survey found that almost one-quarter (22%) of chronic disease practitioners reported that addressing health equity was not a priority in their division.^6^ More information is needed on this topic to develop and promote best practices and monitor improvements over time. The purpose of the current study was to explore the perception of health equity within state chronic disease prevention work units in a sample of four states.

## Methods

### Participant selection

Participants for this case study were selected in conjunction with a related evaluation of the NACDD capacity-building and evidence-based efforts. NACCD is a national organization with over 7000 members whose mission is to improve the health of the public by strengthening state-based leadership and expertise for chronic disease prevention and control in states and at the national level.^17^ In 2015, state chronic disease directors (i.e., the state lead person for chronic disease control efforts) and state chronic disease prevention and health promotion program staff who were also NACDD members were surveyed. Using responses from the survey of NACDD members, states were ranked by overall capacity in evidence-based public health, public health accreditation status, and the preventable burden of disease in each state. From this ranking, four state health departments were invited to participate in the study. To attain variability, two of the four states were identified as higher capacity and two as lower capacity.

Chronic disease directors from each of the four chosen states were invited to participate in a follow-up case study evaluation. This initial interview enabled snowball sampling to identify up to 12 practitioners per state who were also members of NACDD. These practitioners received an invitation to be interviewed.

### Interview guide

The interview guide was based on findings from the initial survey with the focus on evidence-based decision-making. The research team and NACDD partners developed questions and divided them into four main sections: administrative support for evidence-based programs and policies; organizational support for evidence-based interventions; networks and partnerships to support evidence-based decision-making; and health equity. The health equity section (the focus of this article) included questions on programs that have been effective in addressing health equity, recommended steps to improve health equity work, partnerships in health equity efforts, and suggestions for improvement in transdisciplinary work to improve health equity. Interviewers were trained on the purpose of the study, effective interview strategies, and the content of the guide. An incentive for participation consisted of a $25 donation to a nonprofit organization from a list of options. This study was approved by the Washington University in St. Louis Institutional Review Board of the Human Research Protection Office.

### Analysis

Each interview was digitally audio recorded and professionally transcribed. Transcripts were analyzed in NVIVO, v11, using methods consistent with qualitative research standards. First, after reviewing a sample of transcripts, a codebook was developed. Research team members were trained for consistency by coding a sample of transcripts together and discussing discrepancies. Using a constant comparative coding technique,^18^ the remaining transcripts were coded, and then recoded as additional topics were added. Half (*N*=13) were coded by two members of the research team and analyzed for reliability and reduction of positionality and personal bias while coding. Inconsistencies were reconciled with a third reviewer. Coded comments were synthesized into overall themes, and these main themes were further subdivided and categorized.

## Results

All interviews occurred through telephone between December 2016 and May 2017. The number of people interviewed per state ranged from 5 to 8 for a total of 27 chronic disease-related staff participating. The interviews took an average of 60 min (range=21–77 min). Participants averaged 8 years working in a state health department and 13 years working in public health. Those interviewed supervised an average of four staff members and worked in a unit that employed an average of 52 people. Responses to interview questions fell into three broad, inter-related categorical themes that were representative of comments from participants in all four states.

### Thematic results

#### Theme 1: Integration

Interviewees from all of the states mentioned that they were already doing work related to health equity, but to varying degrees and with varied success. Their comments on integrating health equity into state chronic disease prevention programs and the examples they provided were both tangible and intangible. Tangible examples were those that were more evident and formalized. One state has healthy equity integrated into its mission. Other respondents indicated that aspects of health equity were infused in strategic planning efforts. Two of the states represented in this case study had separate divisions for health equity, although a participant in one of the states noted its ineffectiveness due to low funding and lack of adequate staff. All states had some sort of training and technical assistance on health equity, but participants reported that they were not utilized to their fullest extent. One participant noted that even though training exists, there is a misconception about what health equity really is and that a widely accepted universal definition is lacking.

Another tangible example of integrating health equity into chronic disease prevention that was elicited through the interviews was to do so through policies. One example mentioned was a state resolution to create a collaborative community of department and other representatives dedicated to addressing health equity issues. A departmental policy requiring a special part of program planning dedicated to the steps that can be taken to achieve health equity would keep it a priority across programs.

It's actually being built into the funding mechanisms that health equity is something that has to be done. It's not something that is optional.

Last, representatives in all four states mentioned funding, both internal and external. They cited funding needs for training, staff, and collaboration related to increasing the emphasis on health equity efforts within the division and department. It was also noted that even though some funders emphasize the issue, most external funders could do a better job prioritizing evidence-based programs and strategies that address equity.

In addition to tangible examples of health equity integration, there was also discussion of less concrete ways that it is, or could be, supported in state chronic disease prevention. For example, culture within the division—both in leadership and staff—was mentioned as an important influence of prioritizing health equity. For one state that has been addressing health equity for several years, the culture has been slow to evolve in levels of support of the work. Now, health equity is seen as a buzzword that people are tired of because they think they know and are already working on it. Another participant noted that departmental culture is not supportive of health equity because of the misconception of the concept in general.

We need to spend more time talking with each other about why or why not we're supportive of the idea of health equity. How can we talk about it to develop a shared understanding that it's about fairness and it doesn't mean taking something away from someone else so that someone could have more than they have.

Leadership support was also cited as a key element in effective integration of health equity. Participants in all states indicated that top-level support was critical to success, and representatives from one of the states included in the case study praised their leadership team for this.

Our executive leadership team is very supportive and attentive to health equity issues. Success is due to leadership support and focus.

#### Theme 2: Collaboration

Collaboration was a main theme that was elucidated in the interviews. Many respondents mentioned that the root causes of health inequity are complex and connected to social determinants of health. To effectively address health equity and address these determinants, chronic disease prevention practitioners need to collaborate with a broad array of partners. Representatives in all states mentioned partnering with tobacco cessation efforts (e.g., Quitlines) to ensure equitable uptake. Other partners that were noted included food banks, mental health, department of disabilities, minority health, transportation, education, and interpretation services. Even though these partnerships exist, several respondents mentioned that there is a lack of coordination and communication among divisions and that mandatory report-outs would help inform health equity efforts in a more comprehensive way. Many representatives also mentioned that the way in which programs are funded could be a barrier to working together, especially for projects and programs addressing more upstream prevention efforts such as housing or education.

I think something that could be done is addressing some of the more upstream causes of health inequity and health disparities. But, unfortunately, again, it comes back to the funding and what we're funded to do.

Partners in the community were also noted as critical to impacting health equity. They mentioned that development of sustainable relationships with community organizations can make local efforts more effective, but this is not a consistently applied strategy. Community partners may also help practitioners find out where disparities exist at a local level so that there can be more targeted efforts. Several respondents noted that there is a need for including nontraditional partners that are trusted within communities to help with planning and implementing interventions. Local chapters of advocacy agencies (e.g., American Health Association) or local coalitions were mentioned as potential partners to address health equity.

If our partner is the community-based organization, they would have a better sense of what would work than the State Health Department would, and they could advise us and we could work together.

#### Theme 3: Measurement

The third main theme that emerged from the interviews was the lack of data needed to adequately address health equity. The majority of respondents mentioned that data on specific population groups would help drive prioritization of efforts. Gaps exist in what are collected, and as one participant asked “How do we address health equity if [they] really don't know what the inequities are?” Continued surveillance and mandatory reporting of data relating to disparate groups were suggested strategies to address these measurement issues. Additionally, the slow processes of public health data collection and publication were mentioned as barriers to planning and evaluating interventions.

Our entire public health data system suffers from inability to report rapidly and meaningfully on racial and ethnic health disparities. And having more real time information around that would be very, very helpful.

Measurement was also discussed related to showing impact of health equity efforts. Participants noted that lack of data makes program evaluation and demonstration of effectiveness challenging. This also makes it difficult to build evidence so that successful programs could be replicated to broaden reach and ultimately have greater impact.

How do we monitor or audit our work in a sense of its level of effectiveness on the questions of equity?

Several respondents also noted that there is a gap in data not only for planning and evaluation but also for dissemination. Local data presented in a way that is concise and understandable can be used to garner support from a wide range of stakeholders in disparate communities. Community members, organizations, and policy makers were all suggested as groups that would benefit from such information. These data could be used for increasing awareness within communities as well as reporting back after programming or interventions to show impact.

There is a need for information, data, and knowledge about the communities with which they can better tailor messages and engage with communities, legislators, boards, and decision makers to affect change.

## Conclusions

The information from these interviews is helpful in understanding existing health equity efforts within state chronic disease prevention work units and suggests some recommendations for improvement. It was apparent from the interviews that work related to addressing health equity was not absent, but it did not exist to the extent that would have maximum impact. Efforts were reported as fragmented and at times unknown. Having more specific data on health inequities and their root causes could help better define and integrate health equity into chronic disease prevention efforts.

There appears to be a gap between the focus on health equity within national advocacy and public health organizations and actual public health practice. This may be due, in part, to the complexity in the root causes of health inequities. Social determinants of health that cause these inequities (such as access to affordable housing, safe neighborhoods, quality schools, and transportation options) are not traditionally included within the realm of public health or chronic disease prevention.^6^ Surveillance of these factors as related to disease rates and behavioral risk (e.g., spatial epidemiology) may help support targeted initiatives for chronic disease prevention and control.

Although there may be increased awareness of health inequities,^9,11,13,14^ there may be a gap in knowledge about ways to address them and a lack of capacity and political will to do so. The participants in this study indicated that collaboration with interdepartmental and external partners provides expertise and insight into addressing the social determinants to improve chronic disease prevention as well as resource sharing and reduction of duplication of efforts. This is not always easy or feasible due to siloed funding and programming at the state level. Another important aspect of collaboration mentioned by participants is developing and maintaining partnerships with community agencies. Community agencies are well suited to assist with access to the disparate populations that are targets of chronic disease prevention efforts. Another benefit of collaboration, both internally and externally, is that there is evidence to support increased innovation and effectiveness in problem solving with diverse teams.^19^ A 2018 report by the Association of State and Territorial Health Officials emphasizes the need for collaborations with sectors outside of health, such as agriculture, economics, education, housing, justice, and transportation, to advance healthy equity.^20^ State offices of minority health and health equity should be equipped with the capacity to facilitate and coordinate multisector efforts.

There is a need for better and timelier data that identify where chronic disease-related inequities exist in states and communities. Innovative ideas about surveillance and measurement are needed. For example, the Office of Performance, Strategy, and Budget of King County, Washington, developed a new assessment system in their Determinants of Equity Baseline Project. They developed a dashboard to assess a set of key measures that are reflective of the county's conditions across determinants to quantify inequities in a new way and help track progress over time.^21^ The data on inequities can also be layered with chronic disease data, which can be helpful in resource allocation. Innovations such as the King County project should be shared and adapted to other populations.

In addition to tracking of progress with data, there is a need for tracking health equity work within agencies and disseminating information of existing and planned efforts. Several participants noted that this is lacking and a coordinated effort and agency-wide communication would be beneficial. The model set forth by Public Health Accreditation Board can be used for monitoring and evaluating these efforts as disclosure of department efforts related to achieving health equity is required.^16^

Chronic disease practitioners in this study also noted the need for tools and techniques to easily analyze data related to social determinants and effectively disseminate findings to a wide audience. Policy makers and other stakeholders who make key decisions about prevention funding and programming are a particularly important audience for this information.

Our findings suggest that another way to improve state chronic disease response to health equity is to formalize it. Requiring that health equity be included in agency mission statements, reporting, funding, data collection, and training could be ways to operationalize key aspects of health equity more fully. Formalizing health equity in this way will ensure that it is a sustained priority, yet this remains a challenge. In a recent study, Furtado et al. noted that few chronic disease work units have been able to do this because of lack of adequate dedicated resources and staff.^5^ At the same time, many state health departments have issued reports of health inequities, included health equity in their strategic plans, and taken steps to achieve health equity.^22–24^ Even though these reflect great progress, best practices are not yet universal and not always well integrated with the work of chronic disease units.

Making health equity a documented part of chronic disease prevention practice may also lead to increased awareness and a supportive culture within and beyond departments. As indicated in our findings, leadership is important to increased commitment to equity. Leaders can play a significant role in both formalization of health equity as an underlying principle in their work and in fostering a culture where the health equity lens is always in focus. Tools have been recently developed to help public health leaders identify the skills, organizational practices, and infrastructure necessary to achieve health equity.^25,26^

There are limitations inherent with qualitative research. First, we only interviewed practitioners in four states and results may not be generalizable to all states. Expanding this work to be representative of more states and territories would be beneficial for creating a baseline of current health equity needs in state chronic disease prevention. Second, health equity efforts were described by only a sample of people within the states and the information may not be representative of all parts of the state health departments. Third, this case study only explored state-level practitioners. Local health department staff and organizational partners may have different perceptions and are critical given that they connect more closely with communities of focus. Last, our data were limited to information and perceptions from qualitative interviews, and future studies should be triangulated with other data sources for a broader scope of this topic. In spite of these limitations, this case study is a valuable contribution to the health equity literature and chronic disease prevention efforts.

## Abbreviation Used

NACDD: National Association of Chronic Disease Directors
